# Lower common pathway location detected by cryoablation of atrioventricular nodal reentrant tachycardia of the common variety

**DOI:** 10.1002/ccr3.2392

**Published:** 2019-10-06

**Authors:** Kaoru Okishige, Takatoshi Shigeta, Rena A. Nakamura, Tatsuhiko Hirao, Hiroshi Yoshida, Yasuteru Yamauchi

**Affiliations:** ^1^ Heart Center Japan Red Cross Yokohama City Bay Hospital Yokohama Japan

**Keywords:** cry‐catheter ablation, lower common pathway of atrioventricular nodal reentrant tachycardia

## Abstract

As different from radiofrequency current energy, cryofreezing energy is able to provide reversible effects on cardiac tissue, called “cryomapping,” which enables us to predict the effects of a subsequent application of ablative energy. Cryomapping is able to delineate the anatomical location of the lower common pathway of atrioventricular nodal reentrant tachycardia.

## Graphical abstract

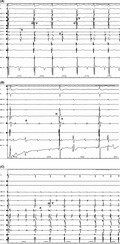

## 1 INTRODUCTION

Cryoablation was performed at infero‐posterior Kock's triangle area in patient with atrioventricular nodal reentrant tachycardia (AVNRT). When ice‐mapping was undertaken, transient AV dissociation was provoked during AVNRT. 1:1 AV conduction was regained immediately after terminating ice‐mapping. When cryoablation catheter was performed at lower site, slow pathway could be completely eliminated.

The concept of a lower common pathway has been utilized for the sake of explaining phenomena of atrioventricular (AV) block without recording a His electrogram and has been a long‐standing controversy of AV nodal reentrant tachycardia (AVNRT).^1^, ^2^, ^3^ Whether or not the lower common turnaround of the circuit is located within the AV node with a region of AV nodal tissue bridging it to the His bundle remains questionable.^4^ The lower common pathway is defined as the conduction path between the distal turnaround point of the AVNRT circuit and His bundle.^5^ The absence of a His bundle depolarization in the blocked beats was considered evidence of intranodal, lower common pathway block or block occurring between the AV node and His bundle.^6^, ^7^ However, Man et al demonstrated that the level of AV block is always below the AV node due to functional infranodal block, even when a His bundle potential is not present in the blocked beats.^3^

Cryoablation is a novel approach for treating AVNRT, as part of the management of drug‐refractory symptomatic AVNRT.^8^, ^9^ Cryoenergy has evolved as a safe and effective alternative for catheter ablation of arrhythmogenic substrates. The therapeutic effects of the cryoenergy application may be predicted by creating reversible conduction block at a target temperature of −30°C allowing verification of the target site before a permanent lesion is produced (“cryomapping”).^10^ We present a case highlighting the location of the lower common pathway in a patient with AVNRT.

## 2 CASE REPORT

**Figure 1:**
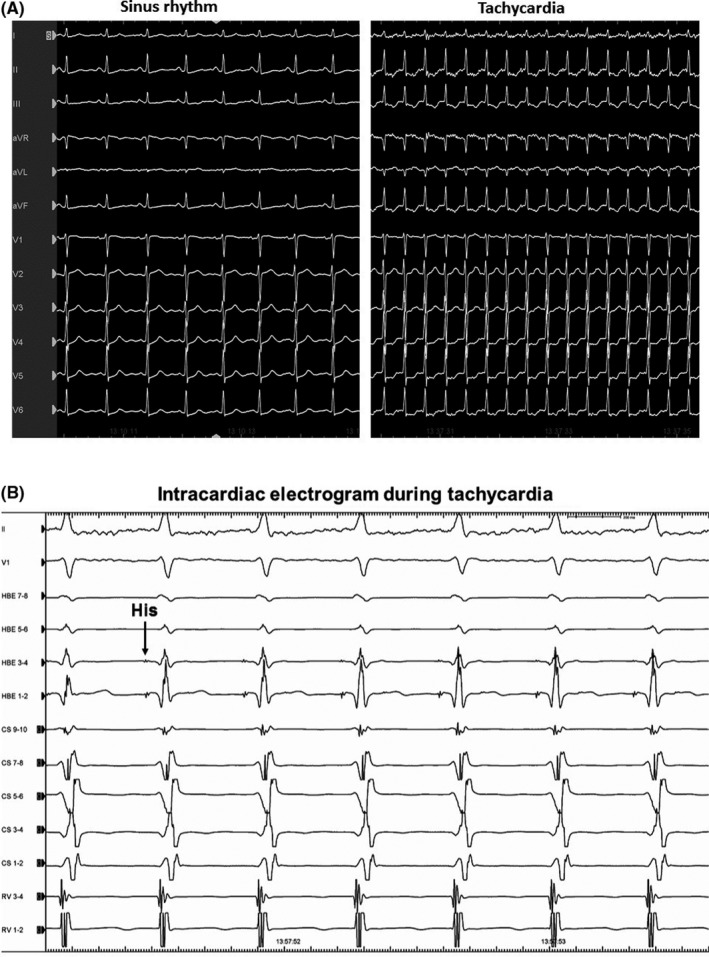
A, 12‐lead ECG during sinus rhythm (NSR) and tachycardia. B, Intracardiac electrograms during tachycardia. His, His bundle electrogram

**Figure 2:**
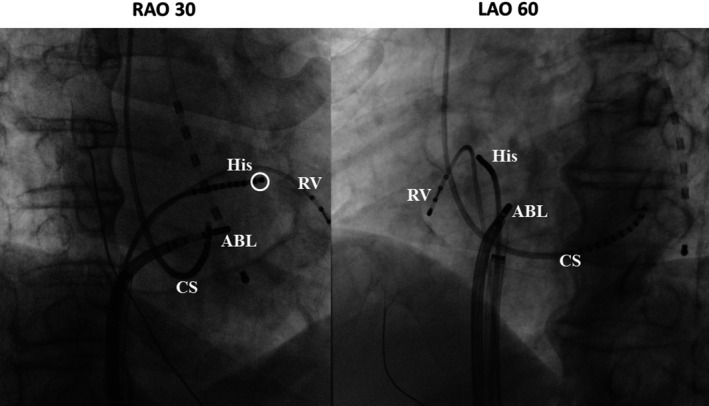
Fluoroscopic image of the electrode catheters. The location of the tip electrode at the cryoablation (ABL) site indicates the exact portion of the lower common pathway. Open circle indicates the His bundle recording electrode location. ABL, ablation catheter. CS, coronary sinus; His, His bundle; LAO, left anterior projection; RAO, right oblique projection; RV, right ventricle

**Figure 3:**
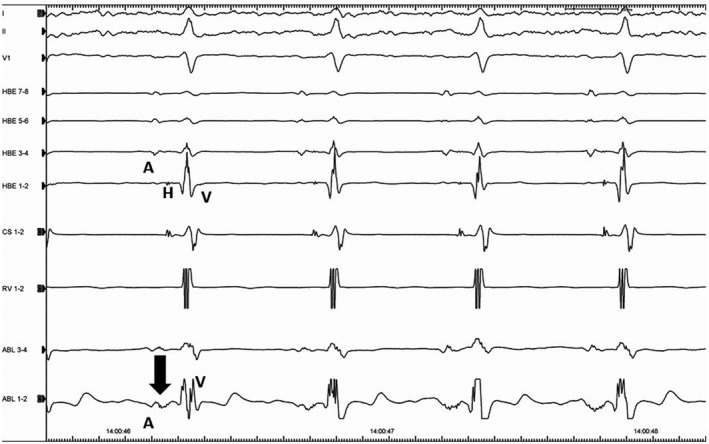
Intracardiac electrograms at the ablation site during sinus rhythm. The arrow indicates the atrial electrogram showing a so‐called “ slow pathway potential” recorded from the tip electrode of the cryoablation catheter. A,atrial electrogram; H, His bundle electrogram; V,ventricular electrogram

**Figure 4:**
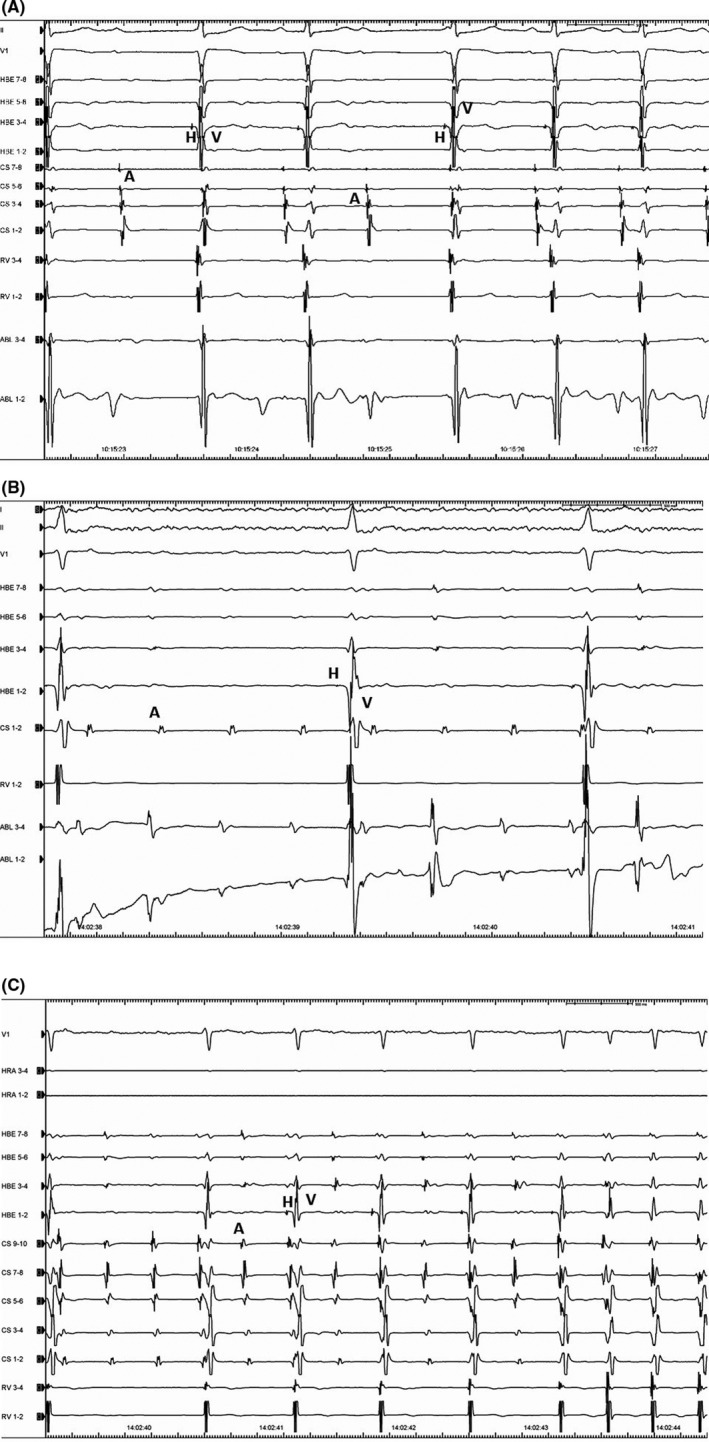
Panel A, Transient AV block was induced when approximately 30 s had passed since initiating the cryomapping at a temperature of −30°C when the cryoablation catheter was positioned in the anterosuperior portion of Kock's triangle. Panel B, Further degree of AV block was provoked during cryomapping at a temperature of −30°C for 30 s when the cryoablation catheter was moved posteriorly downward from the first site. Panel C, 1:1 AV conduction could be obtained immediately after terminating the cryomapping. A, atrial electrogram, H, His bundle electrogram, V, ventricular electrogram

A 54‐year‐old woman was referred to treat a narrow QRS complex tachycardia, which could be terminated by 20 mg of intravenous adenosine triphosphate. Written informed consent was obtained from the patient for the electrophysiological study (EPS) and ablation. All antiarrhythmic drugs were discontinued for five half‐lives before the procedures. Two quadripolar electrode catheters were positioned in the high right atrium and right ventricular apex. Two 10‐pole electrode catheters were introduced into the coronary sinus (CS) and located at the His bundle recording site. Bipolar electrograms were filtered between 50 and 400 Hz and recorded along with the surface electrogram using a polygraph (Prucka, General Electric Company). A single atrial programmed extrastimulation was able to induce and atrial burst pacing was able to terminate this sustained AVNRT without the administration of isoproterenol (Figure 1A, B). The tachycardia could be reproducibly induced and terminated by programmed atrial stimulation, and the induction was always related to a jump‐up of the AH interval. We performed ventricular burst pacing during this tachycardia, and an atrial response to the ventricular pacing with entrainment exhibiting a V‐A‐V sequence upon cessation of the ventricular pacing was observed. The mean AVNRT cycle length was 314 ± 28.4 ms A cryoablation catheter (Freezor Xtra, Medtronic) was positioned at the apex of Kock's triangle (Figure 2), and a so‐called “slow pathway potential” was recorded from the tip electrode of the cryoablation catheter without recording a His bundle electrogram (Figure 3). When cryomapping at a temperature of −30°C was performed at that site during sustained AVNRT, transient AV block during the sustained AVNRT was provoked lasting for 4 seconds when 19 seconds has passed since the start of the cryomapping (Figure 4A). A further degree of AV block was induced lasting for 9 seconds without terminating the AVNRT, and it occurred 27 seconds after initiating the cryomapping (Figure 4B). However, the AVNRT could not be terminated during the cryomapping for 60 seconds. When we stopped performing the cryomapping, 1:1 AV conduction over the AV node was regained 2 seconds after terminating the cryomapping (Figure 4C). We moved the cryoablation catheter infero‐posteriorly downward by 2 mm where the cryomapping was able to repeatedly terminate the AVNRT without impairing the AV nodal conduction. Cryoablation at a temperature of −80°C for 240 seconds was performed three times at that site resulting in the successful complete elimination of the AVNRT without any adverse events.

## 3 DISCUSSION

We report a case in which the location of the lower common pathway was detected in a patient with AVNRT using cryofreezing energy, which was able to induce transient effects on the conduction system depending on the nadir temperature during the freezing. To the best of our knowledge, this is the first case to localize the portion of the lower common pathway. The lower common pathway is defined as the conduction pathway between the distal turnaround point of the AVNRT circuit and His bundle. Several reports have demonstrated the cure of AVNRT cases using radiofrequency current (RF) energy, in which an “upper common pathway” and “lower common pathway” were detected.^2^, ^11^ However, they did not locate the site of the lower common pathway except for in one experimental study.^12^ We considered that only cryomapping could make the localization of the lower common pathway possible, because cryofreezing energy can provoke transient effects on the cardiac conduction system.^13^ The location of the lower common pathway was detected in the anterosuperior portion of Kock's triangle where cryofreezing and RF energy were able to successfully eliminate the slow pathway. Whether the lower common pathway was involved in the intranodal area or not could not be determined in the present case, because pharmacological examination such as with an atropine loading test was not performed, which would have enabled the localization of the lower common pathway, intranodal or infranodal. The slow pathway seemed to be located posteriorly, lower than the site of the lower common pathway considering the observations from the fluoroscopic image.

AV block during sustained AVNRT is often seen at the onset of a very fast AVNRT due to the relatively long refractory period below the circuit. Therefore, block during AVNRT does not necessarily define a lower common pathway. However, 1:1 AV conduction was maintained all throughout the sustained AVNRT, and intermittent AV block could be observed only during cryomapping.

Although previous reports concluded the level of the AV block based only on the presence or absence of a His bundle potential in the blocked beats,^6^, ^7^ Man et al insisted that it was intra‐Hisian block regardless of the status of the His bundle potential in the blocked beats.^3^ In up to one‐third of patients with AVNRT, the lower turnaround point of the circuit is within the His bundle, arguing against an intranodal circuit of AVNRT.^14^

## 4 CONCLUSION

The exact location of the lower common pathway was proven by the response to cryomapping during sustained AVNRT. Cryofreezing energy is useful in terms of not only closely analyzing the physiology of the AV node but also curing arrhythmias that involves the AV node as a requisite part of the arrhythmia circuit.

## CONFLICT OF INTEREST

None declared.

## AUTHOR CONTRIBUTIONS

Kaoru Okishige, MD, FACC, FHRS, FJCS, Takatoshi Shigeta, MD, Rena A. Nakamura, MD, Tatsuhiko Hirao, MD, Hiroshi Yoshida, MD, Yasuteru Yamauchi, MD Kaoru Okishige: performed cryoablation for this patient and wrote this manuscript as a chief scientist. Takatoshi Shigeta: performed catheter ablation of this case with Okishige. Rena A. Nakamura: took care of this patient during hospitalization. Tatsuhiko Hirao: performed catheter ablation with Okishige. Yasuteru Yamauchi: manipulated the computer system and analyzed the data regarding intracardiac electrocardiography during this ablation case.
